# Structural basis of nirmatrelvir and ensitrelvir activity against naturally occurring polymorphisms of the SARS-CoV-2 main protease

**DOI:** 10.1016/j.jbc.2023.103004

**Published:** 2023-02-10

**Authors:** Gabriela Dias Noske, Ellen de Souza Silva, Mariana Ortiz de Godoy, Isabela Dolci, Rafaela Sachetto Fernandes, Rafael Victório Carvalho Guido, Peter Sjö, Glaucius Oliva, Andre Schutzer Godoy

**Affiliations:** 1 Sao Carlos Institute of Physics, University of Sao Paulo, Sao Carlos, Brazil; 2 Drugs for Neglected Diseases Initiative (DNDi), Geneva, Switzerland

**Keywords:** SARS-CoV-2, ensitrelvir, nirmatrelvir, resistance, COVID, paxlovid, xocova, main protease, mpro, drug, mutation, structure, x-ray, DMSO, dimethyl sulfoxide, M^pro^, Main protease

## Abstract

SARS-CoV-2 is the causative agent of COVID-19. The main viral protease (M^pro^) is an attractive target for antivirals. The clinically approved drug nirmatrelvir and the clinical candidate ensitrelvir have so far showed great potential for treatment of viral infection. However, the broad use of antivirals is often associated with resistance generation. Herein, we enzymatically characterized 14 naturally occurring M^pro^ polymorphisms that are close to the binding site of these antivirals. Nirmatrelvir retained its potency against most polymorphisms tested, while mutants G143S and Q189K were associated with diminished inhibition constants. For ensitrelvir, diminished inhibition constants were observed for polymorphisms M49I, G143S, and R188S, but not for Q189K, suggesting a distinct resistance profile between inhibitors. In addition, the crystal structures of selected polymorphisms revealed interactions that were critical for loss of potency. In conclusion, our data will assist the monitoring of potential resistant strains, support the design of combined therapy, as well as assist the development of the next generation of M^pro^ inhibitors.

SARS-CoV-2 is a highly transmissible β-coronavirus (1, 2) with a genome composed of a single RNA positive strand that comprises about 30 kb encoding for 16 nonstructural, four structural, and six accessory proteins (3). The viral replicase codifies two frame shifting open reading frames, ORF1a/ORF1ab, containing 16 nonstructural proteins required for viral replication (4). The SARS-CoV-2 Main protease (M^pro^) or 3C-like protease (3CL^pro^) is a dimeric cysteine protease responsible for the cleavage of the viral polyproteins 1a and 1ab in 11 sites, including its own N and C terminal (5, 6, 7). M^pro^ substrate recognition has unique features and is specific for Gln residue at P1, hydrophobic residues at P2, and small side chains such as Ser and Ala at P1’ (8). The absence of similar sites in human proteases together with the importance of the enzyme for the viral replication makes M^pro^ a primary target for antiviral discovery and development.

Several small molecules were identified as M^pro^ inhibitors that exhibited efficacy in cellular culture including boceprevir, carmofur, MAT-POS-e194df51-1, PF-07321332 (nirmatrelvir), and S-217622 (ensitrelvir) (6, 9, 10, 11, 12, 13, 14). The first oral COVID-19 antiviral from Pfizer, Paxlovid, is a combination of reversible covalent M^pro^ inhibitor nirmatrelvir and ritonavir, a CYP3A4 inhibitor, with safety and efficacy demonstrated in clinical trials, and approved for use by the US Food and Drug Administration in December 2021 (14, 15). In addition, the compound ensitrelvir from Shionogi is a promising noncovalent inhibitor of M^pro^ (11). Currently in phase 3 clinical trials, the compound has shown exciting pharmacokinetics properties, with potential for therapeutic doses to be reached without requirement of CYP inhibitors (11).

Although most polymorphisms are not expected to generate a variant of concern (16), World Health Organization is constantly monitoring the emergence of SARS-CoV-2 mutations, since recent variants have exhibited more transmissible and infectious properties and can affect vaccines effectiveness (17, 18). Moreover, amino acid replacements in the viral protein can impact the catalytic activity of the enzyme and modify the efficacy of inhibitors (19, 20). The *in vitro* effectiveness of nirmatrelvir against variants Alpha (B.1.1.7), Beta (B.1.351), Delta (B1.617.2), Gamma (P.1), Lambda (B.1.1.1.37/C37), and Omicron (B.1.1.529) has already been demonstrated (21). However, none these variants contain mutations that are in the vicinity of the active site. Herein, we evaluated the effect of single mutations in the active site of M^pro^ from circulating polymorphisms on the kinetics and inhibitory constants of nirmatrelvir and ensitrelvir. We also used X-ray crystallography to characterize the structural features of selected polymorphisms. These findings provided key information for predicting and avoiding resistance, designing the next generation of inhibitors, and raised important considerations for combination therapies.

## Results

**Figure 1:**
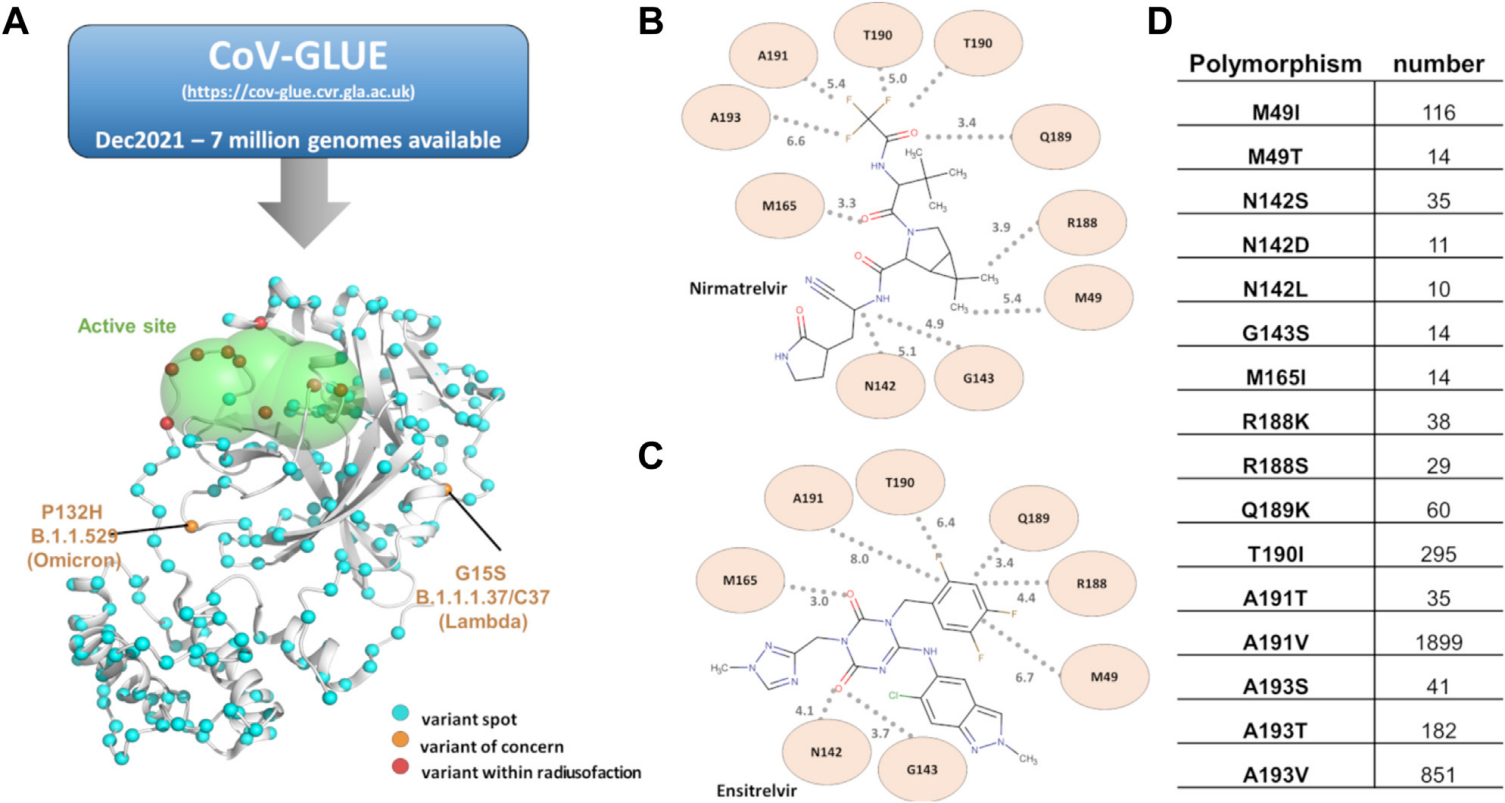
**SARS-CoV-2 M**^**pro**^**amino acids polymorphisms identified in the genomic database.***A*, M^pro^ model showing variant spots identified in the genomic database, using a threshold of n ≥ 10. The Cα of identified variants spots are shown as *cyan spheres*, while *red spheres* are those variants within a radius of 8 Å ligands. *Orange spheres* show spots for variants of concern. Active site of M^pro^ is colored with a *green blob*. *B*, 2D plot of nirmatrelvir showing the distance and closest contacts with selected polymorphisms. *C*, 2D plot of ensitrelvir showing the distance and closest contacts with selected polymorphisms. *D*, panel containing polymorphism name, and substitution, and number of individuals identified (n) in a pool of 7 million SARS-CoV-2 genomes cataloged at CoV-GLUE by December 2021.

For this study, we selected active site polymorphic versions of M^pro^ that have been already identified in circulation. For that, we assessed sequencing data available from GISAID hCoV-19/SARS-CoV-2 sequences database (22) (containing approximately 7 million genomes as of December 20, 2021) in CoV-GLUE (http://cov-glue.cvr.gla.ac.uk) relative to M^pro^. We identified 389 distinct polymorphisms in M^pro^ with a n ≥ 10 individuals. From those and based on available structural information, we selected mutants that were within an 8.0-Å radius of each inhibitor structure, which would allow us to investigate how near active site mutations affect drug effectiveness against the enzyme. We identified 16 polymorphisms, which are summarized in Figure 1, including four key nirmatrelvir contact residues (*e.g.*, M49, G143, M165, and Q189). To investigate the impacts of the polymorphisms on M^pro^ activity and calculate inhibitors constants, we were able to express, purify, and characterize 15 of those mutants. Mutant N142L did not generate any soluble protein and was therefore not characterized further.

**Figure 2:**
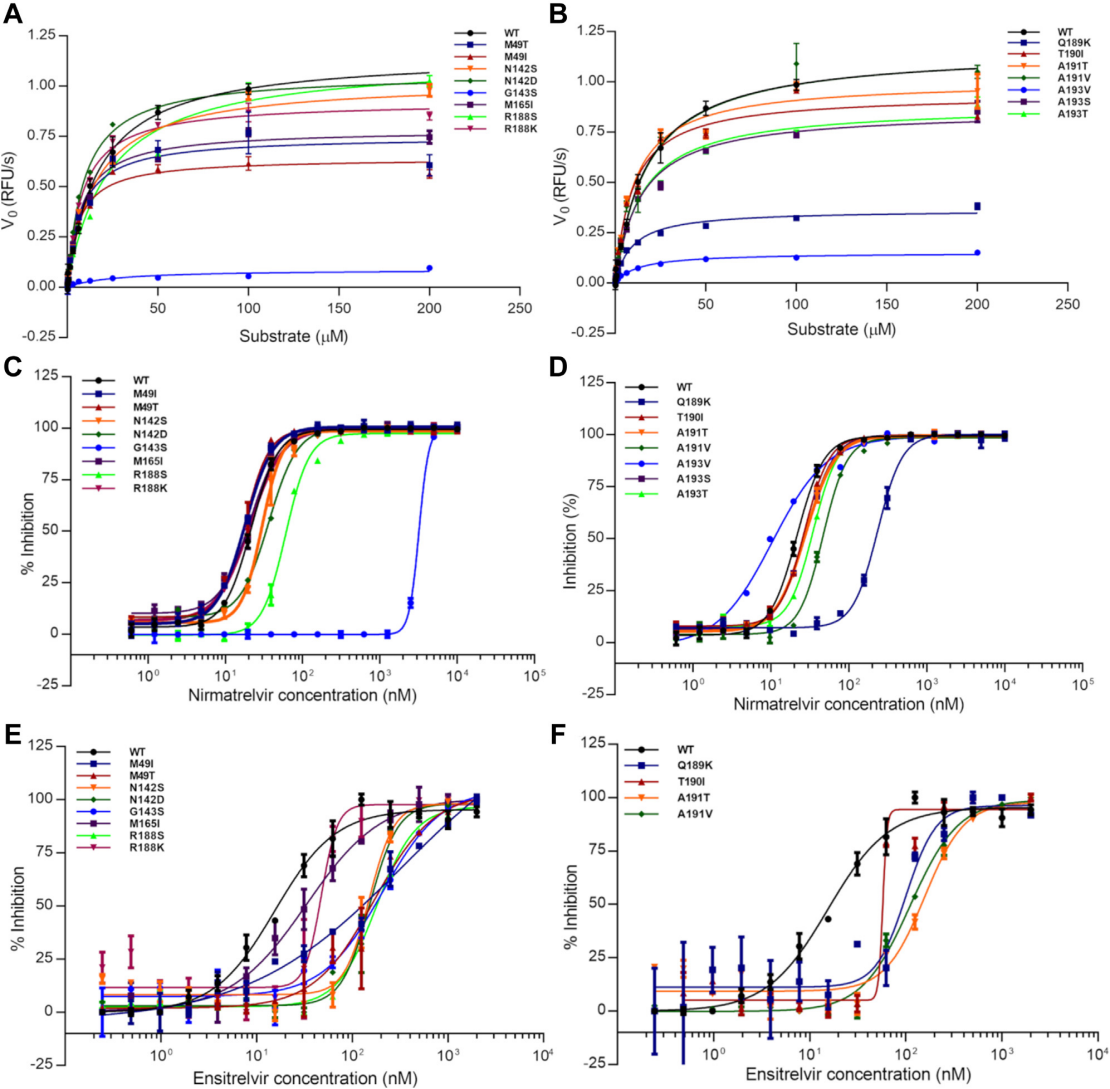
**SARS-CoV-2 M**^**pro**^**polymorphisms kinetics and inhibitions plots.***A*, Michaelis–Menten plots of WT and mutants M49T, M49I, N142S, N142D, G143S, M165I, R188S, and R188K. *B*, Michaelis–Menten plots of WT and mutants Q189K, T190I, A191T, A191V, A193V, A193S, and A193T. *C*, IC_50_ plots determination of nirmatrelvir against WT and mutants M49T, M49I, N142S, N142D, G143S, M165I, R188S, and R188K. *D*, IC_50_ plots determination of nirmatrelvir against WT and mutants Q189K, T190I, A191T, A191V, A193V, A193S, and A193T. *E*, IC_50_ plots determination of ensitrelvir against WT and mutants M49T, M49I, N142S, N142D, G143S, M165I, R188S, and R188K. *F*, IC_50_ plots determination of ensitrelvir against WT and mutants Q189K, T190I, A191T, and A191V. Data in graphs are means ± SD.

All M^pro^ mutants were able to recognize and cleave the fluorogenic substrate. The turnover number (k_cat_) and Michaelis–Menten constant (*K*_M_) were determined for all mutants (Fig. 2), and values are summarized in Table 1 together with the calculated catalytic efficiency (k_cat_/*K*_M_) relative to the wildtype (WT). The WT enzyme showed a *K*_M_ value of 22 ± 2 μM and a *k*_cat_ value of 31 ± 1 s^−1^, which agrees with previous characterizations of M^pro^ for this substrate (16.4 μM and 28 s^−1^, respectively) (7). The mutant panel exhibited *K*_M_ values ranging from 6.4 to 25.4 μM, and relative catalytic efficiencies to the WT that ranged between 3 and 205% (Table 1).

**Table 1:** Kinetic parameters of M^pro^ mutants

Polymorphism	*K*_*m*_ (μM)	k_cat_ (RFU/μM.s)	Relative efficiency
M49I	6 ± 1	16.0 ± 0.6	1.8 ± 0.2
M49T	7 ± 2	19 ± 1	1.8 ± 0.2
N142S	15 ± 3	25 ± 1	1.2 ± 0.2
N142D	9 ± 1	27 ± 2	2.0 ± 0.1
G143S	25 ± 11	1.0 ± 0.1	0.030 ± 0.001
M165I	7.7 ± 0.7	19.6 ± 0.5	1.8 ± 0.1
R188K	8.2 ± 0.9	23 ± 1	2.00 ± 0.08
R188S	22 ± 4	28 ± 2	0.9 ± 0.3
Q189K	9 ± 1	4.5 ± 0.2	0.30 ± 0.09
T190I	10 ± 2	23 ± 1	1.7 ± 0.2
A191T	11 ± 1	25 ± 1	1.7 ± 0.2
A191V	18 ± 3	29 ± 2	1.3 ± 0.3
A193S	14 ± 2	21.5 ± 0.8	1.1 ± 0.2
A193T	14 ± 2	22.1 ± 0.9	1.0 ± 0.2
A193V	13 ± 2	7.5 ± 0.3	0.4 ± 0.1
Wildtype	22 ± 2	30.8 ± 0.8	1.0 ± 0.2

Relative efficiency is the k_cat_/*K*_*m*_ of polymorphisms relative to wildtype M^pro^. Data in table are means ± SD.

**Figure 3:**
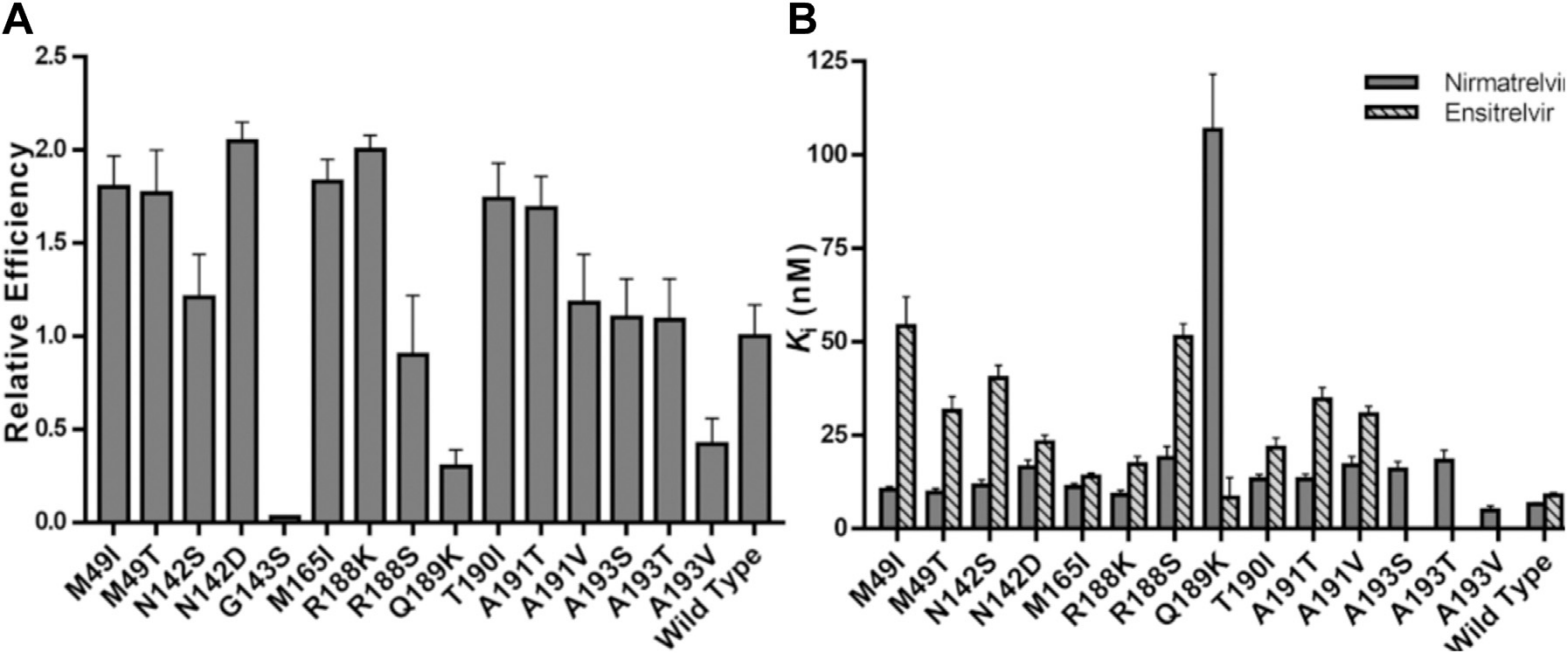
***In vitro* characterization of SARS-CoV-2 M**^**pro**^**mutants.***A*, relative catalytic efficiencies of M^pro^ mutants against FRET substrate. Data in graphs are means ± SD; n = 3. *B*, *K*_i_ determination of nirmatrelvir and ensitrelvir against M^pro^ mutants. Values were calculated using the results obtained in the FRET-based activity assay. For a better visualization, *K*_i_ values of mutant G143S are not represented in this graph. Data in graphs are means ± SD. FRET, fluorescence resonance energy transfer.

Mutants M49I, M49T, N142D, M165I, R188K, T190I, and A191T exhibited a catalytic efficiency significantly higher than the WT, with increments up to 2-fold, with N142D and R188K being the ones with the higher relative catalytic efficiencies (Fig. 3*A*). Mutants N142S, R188S, A191V, A193S, and A193T showed similar relative catalytic efficiencies to the WT (Fig. 3*A*). Mutants G143S, Q189K, and A193V showed diminished relative catalytic efficiencies in comparison with the WT, with a reduction of 33-, 3-, and 2-fold, respectively (Fig. 3*A*).

Next, we evaluated the ability of nirmatrelvir to inhibit WT enzyme and the mutant panel. Our assay confirmed that nirmatrelvir is a nanomolar inhibitor of WT M^pro^, with an IC_50_ value of 0.022 ± 0.004 μM and calculated inhibition constant (*K*_i_) of 0.006 ± 0.0005 μM, in agreement with the previous reported value 0.003 μM for the *K*_i_ (14). Nirmatrelvir also potently inhibited most of mutants in the nanomolar range, with *K*_i_ values ranging from 0.005 to 0.106 μM (Table 2). The only exception was the G143S mutant, where nirmatrelvir *K*_i_ value was 0.96 ± 0.23 μM. Mutants M49I, M49T, N142S, M165I, R188K, T190I, A191T, and A193V showed higher susceptibility to nirmatrelvir, with *K*_i_ values ranging from 0.005 to 0.013 μM, a no more than 2-fold increase in *K*_i_ values relative to the WT (Fig. 3*B*). Mutants N142D, R188S, A191V, A193S, and A193T showed modest diminished inhibitory constants for nirmatrelvir, with 2- to 3-fold increase in *K*_i_ values relative to the WT. Mutants Q189K and G143S exhibited the highest diminished inhibitory constants for nirmatrelvir, showing 16- and 147-fold increase in the *K*_i_ values relative to the WT, respectively (Table 2).

**Table 2:** Inhibition of nirmatrelvir and ensitrelvir against SARS-CoV-2 M^pro^ polymorphisms

Polymorphism	Nirmatrelvir			Ensitrelvir		
	IC_50_ (nM)	*K*_i_ (nM)	*K*_i_ fold increase	IC_50_ (nM)	*K*_i_ (nM)	*K*_i_ fold increase
M49I	17.1 ± 0.5	10.4 ± 0.87	1.60	139 ± 8	54.2 ± 7.8	6.09
M49T	16.9 ± 0.5	9.6 ± 1.15	1.48	73.0 ± 0.3	31.6 ± 3.8	3.55
N142S	29 ± 1	11.5 ± 1.57	1.77	67 ± 2	40.4 ± 3.3	4.54
N142D	32 ± 1	16.45 ± 1.90	2.53	48.0 ± 0.2	23.2 ± 1.9	2.61
G143S	3380 ± 128	960.2 ± 237	147.7	197 ± 0.5	141.3 ± 17	15.9
M165I	19 ± 1	11.1 ± 0.99	1.71	32.0 ± 0.4	13.9 ± 0.9	1.56
R188K	17 ± 1	9.0 ± 1.22	1.38	38 ± 3	17.2 ± 2.1	1.93
R188S	61 ± 3	18.95 ± 3.11	2.92	74 ± 1	51.3 ± 3.5	5.76
Q189K	203 ± 16	106.7 ± 14.9	16.4	38 ± 9	8.3 ± 2.4	0.93
T190I	25 ± 1	13.2 ± 1.38	2.03	44 ± 2	21.6 ± 2.7	2.43
A191T	27 ± 1	13.2 ± 1.43	2.03	67 ± 2	34.6 ± 3.2	3.89
A191V	46 ± 2	16.9 ± 2.45	2.60	48.0 ± 0.1	30.7 ± 2.1	3.45
A193S	27 ± 1	15.8 ± 2.15	2.43	N.D.	N.D.	N.D.
A193T	32 ± 1	18.2 ± 2.75	2.80	N.D.	N.D.	N.D.
A193V	11 ± 2	4.8 ± 1.3	0.74	N.D.	N.D.	N.D.
Wildtype	21.9 ± 0.4	6.5 ± 0.46	1	13 ± 1	8.9 ± 0.71	1

IC_50_ fold increase is relative to the WT. Data in table are means ± SD.

Abbreviation: N.D., not determined.

Next, we evaluated the ability of ensitrelvir to inhibit WT enzyme and the mutant panel. Our assay confirmed ensitrelvir as a potent nanomolar inhibitor of WT M^pro^, with IC_50_ value of 0.013 ± 0.004 μM and calculated *K*_i_ of 0.009 ± 0.0007 μM, in agreement with previous reported values of 0.013 μM for the IC_50_ (12). Ensitrelvir potently inhibited all the mutants in the nanomolar range, with *K*_i_ values ranging from 0.009 to 0.141 μM (Table 2). Mutants M165I, R188K, and Q189K showed higher susceptibility to ensitrelvir, with *K*_i_ values ranging from 0.008 to 0.017 μM, a no more than 2-fold increase in *K*_i_ values relative to the WT (Fig. 3*B*). Mutants N142D and T190I showed modest diminished inhibitory constants for ensitrelvir, with 2- to 3-fold increase in *K*_i_ values relative to the WT. Mutants M49T, N142S, A191T, and A191V showed significantly diminished inhibitory constants against ensitrelvir, with 3- to 5-fold increase in *K*_i_ values relative to the WT. Mutants M49I, G143S, and R188S showed the highest diminished inhibitory constants of ensitrelvir, exhibiting 6-, 15-, and 5.7-fold increase in the relative *K*_i_ values, respectively (Table 2).

For an in-depth understanding of structural basis for the resistance profile of the M^pro^ mutants toward nirmatrelvir and ensitrelvir, we determined the crystal structure of selected M^pro^ mutants in complex with both drugs. For nirmatrelvir complexes, we determined the crystals structures of the WT enzyme, as well as M49I, N142S, G143S, Q189K, A193T, and A193S mutants at 2.1, 1.9, 1.8, 1.6, 2.4, 2.5, and 1.9-Å resolution, respectively. These structures were refined to *R*_work_/*R*_free_ values of 0.19/0.23, 0.20/0.24, 0.23/0.26, 0.21/0.25, 0.22/0.30, 0.22/0.27, and 0.20/0.24, respectively. All crystal structures were obtained in the orthorhombic space group with one dimer of M^pro^ in the asymmetric unit, following the pattern of the seeding samples used, whereas the G143S mutant was solved in the monoclinic space group with a similar crystal packing but with two dimers in the asymmetric unit. Data collection and refinement statistics for the mutants in complex with nirmatrelvir are summarized in Table S2. For ensitrelvir, we determined the crystal complexes of WT and the M49I mutant at 2.3 and 2.0 Å and refined those to *R*_work_/*R*_free_ 0.22/0.27 and 0.22/0.25, respectively. Data collection and refinement statistics for WT and the M49I mutant in complex with ensitrelvir are summarized in Table S3.

**Figure 4:**
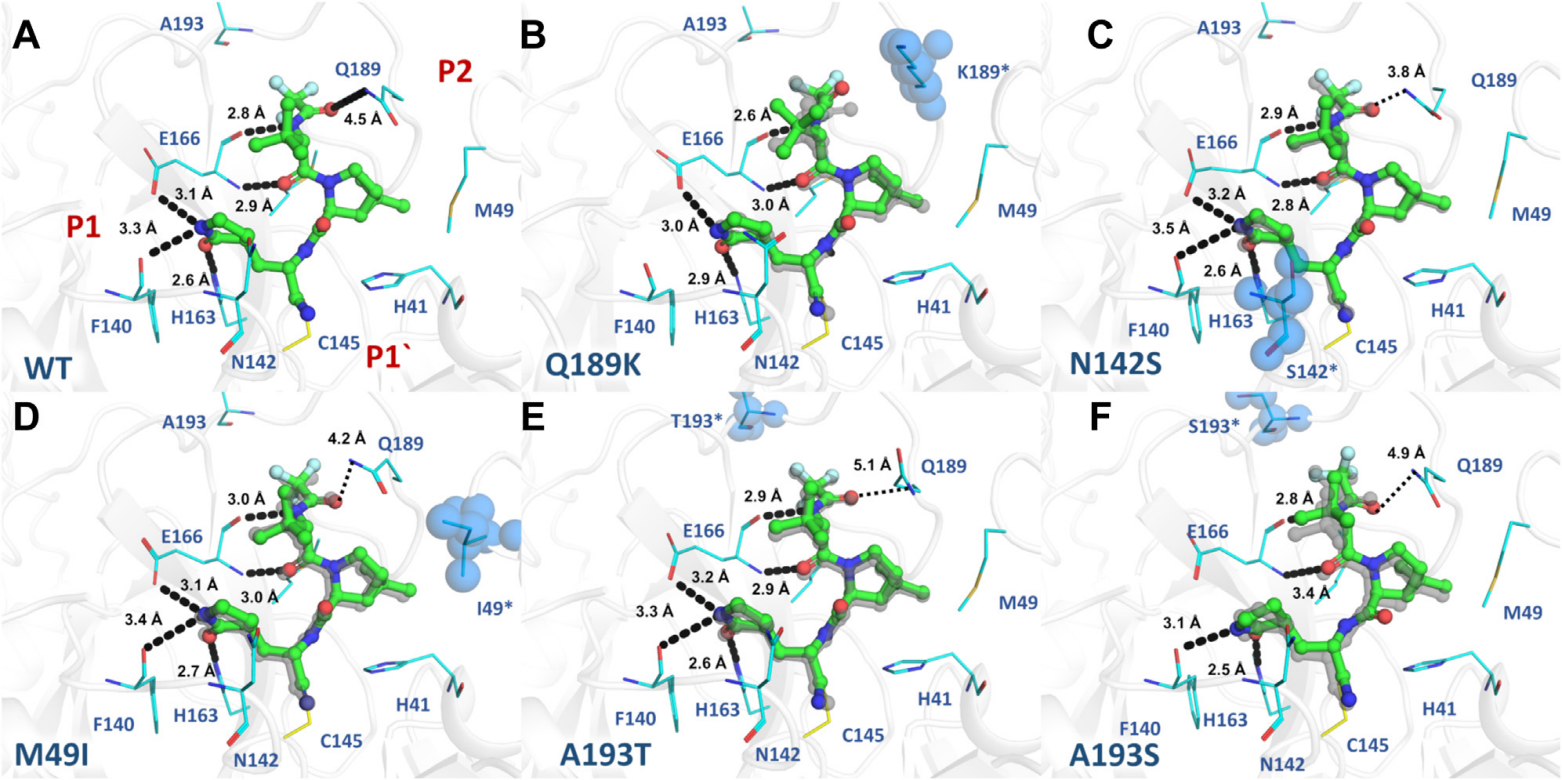
**Crystal structures of M**^**pro**^**mutants in complex with nirmatrelvir.** M^pro^ is displayed as cartoon and colored in *gray*; mutant residues are shown as *spheres* and colored in *blue*. Nirmatrelvir is shown as *ball* and *stick* and colored in *green*. Selected residues are shown as *lines* and colored in *cyan*. Substrate-binding subsites are labeled in *red*. *A*, M^pro^ WT (PDBid 8DZ2). *B*, mutant Q189K (PDBid 8DZ6). *C*, mutant N142S (PDBid 8E26). *D*, mutant M49I (PDBid 8E25). *E*, mutant A193T (PDBid 8DZA). *F*, mutant A193S (PDBid 8E1Y). WT crystal structure is aligned with each M^pro^ mutant, displayed as *gray transparent sticks*. Polar contacts are showed as *black dashes*.

In general, nirmatrelvir exhibited a similar binding mode in all mutants, maintaining most of the key interactions with M^pro^-binding site residues. For all, the electron densities around C145 clearly indicates the presence of a covalent bond between the nitrile carbon of nirmatrelvir and the Sγ atom of C145 (∼1.8 Å). The WT structure in complex with nirmatrelvir shows key interactions between protein and ligand, including three polar contacts between the pyrrolidone group and amino acids F140 (3.3 Å), H163 (2.6 Å), and E166 (3.1 Å), as well as other two hydrogen bonds between the tert-butyl moiety of nirmatrelvir and E166 N (2.9 Å) and O (2.8 Å), and a salt bridge between nirmatrelvir O atom with Q189 side chain NE2 (4.5 Å) (Fig. 4).

In nirmatrelvir WT complex, the S2 subsite is occupied by the dimethyl-cyclopropyl-proline substituent, which interacts mainly by hydrophobic interactions with the main chains of R188 and the side chains of H41, M49, M165, and Q189 (Fig. 4). The same pattern seems to be maintained for all mutant crystal complexes, including mutant M49I (Fig. 4*D*). The structure of the Q189K mutant in complex with nirmatrelvir showed that the replacement of Q189 with lysine resulted in the disruption of a salt bridge between the NE2 of glutamine and the O atom from the carbonyl group of nirmatrelvir, dislocating the ligand position compared with the WT crystal structure and causing the loss of a productive hydrogen contact between the pyrrolidone moiety and the O from amino acid F140 in S1 (Fig. 4*B*).

The tert-butyl substituent at P3 position remained solvent exposed for all mutants, as observed for the WT crystal structure. The S4 subpocket accommodates the trifluoroacetamide substituent at position P4, where the main chain oxygen of the E166 residue makes a hydrogen bond with nirmatrelvir amide nitrogen N1 (Fig. 4*A*). For both crystal structures with mutations of residue 193, A193S and A193T, even though being the most distant mutations of the active site, the additional steric volume and polar features of the serine and threonine side chains caused a displacement in the ligand binding mode in comparison with the binding mode to the WT (Fig. 4, *E* and *F*). The binding modes of nirmatrelvir to both A193S and A193T mutants were similar, but the displacement is more severe in A193S, causing the disruption of pyrrolidone hydrogen bound with E166 (Fig. 4*E*).

**Figure 5:**
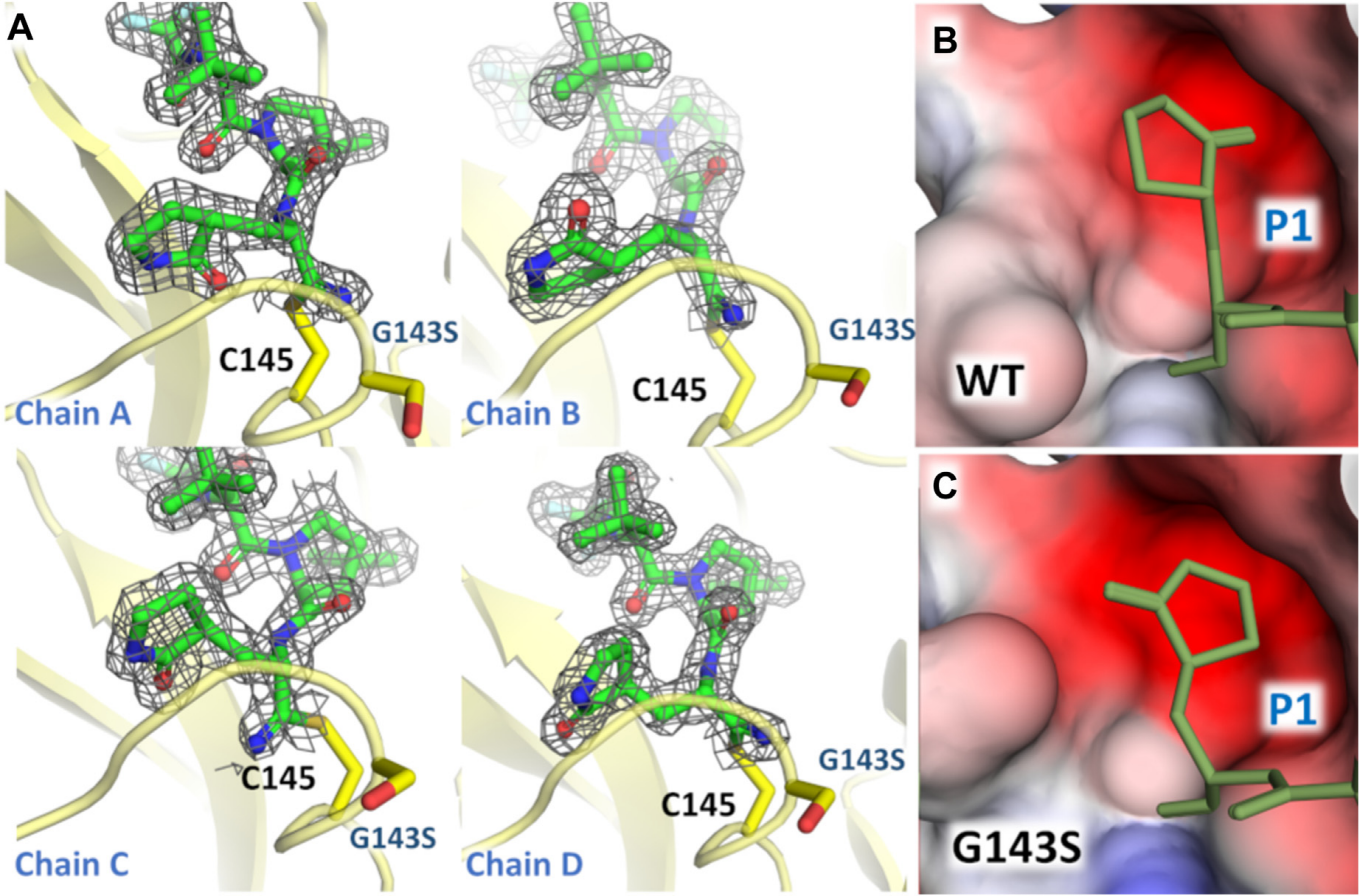
**Crystal structure of G143S M**^**pro**^**mutant in complex with nirmatrelvir.***A*, chains A–D of G143S M^pro^ in complex in nirmatrelvir highlighting the different ligand conformations. M^pro^ is shown as *yellow cartoon*. Residues G143 and C145 are shown as *sticks* and colored in *yellow*. For nirmatrelvir, the 2mFo-DFc electron density contoured at 1.0σ is represented in *gray*. *B*, surface charge representation of S1 subpocket for WT M^pro^, showing the conformation of the pyrrolidine group. *C*, electrostatic charge calculated with APBS (43), projected on surface representation of S1 subpocket for G143S M^pro^ showing the conformation of the pyrrolidine group. For (*B*) and (*C*) nirmatrelvir is shown as *lines* and colored in *yellow*.

C145 and G143 residues are responsible for stabilizing the oxyanion hole, where the backbone nitrogen interacts through hydrogen bonds (Fig. 4*A*). For the mutant G143S complex, we observed that the pyrrolidone group from nirmatrelvir that occupies the S1 pocket appears in four distinct orientations, one to each chain in the two dimmers composing the asymmetric unit (Fig. 5). The mutant G143S complex revealed that the substitution of a glycine residue with a serine changed the charge distribution around S1 subpocket, causing multiple conformations of the pyrrolidone moiety (Fig. 5). As a crystallographic consequence, the multiple conformations of the pyrrolidone caused the break of one of the orthorhombic 2-fold symmetry axis, shifting this crystal to a monoclinic space group with cells like the above (Table S2).

**Figure 6:**
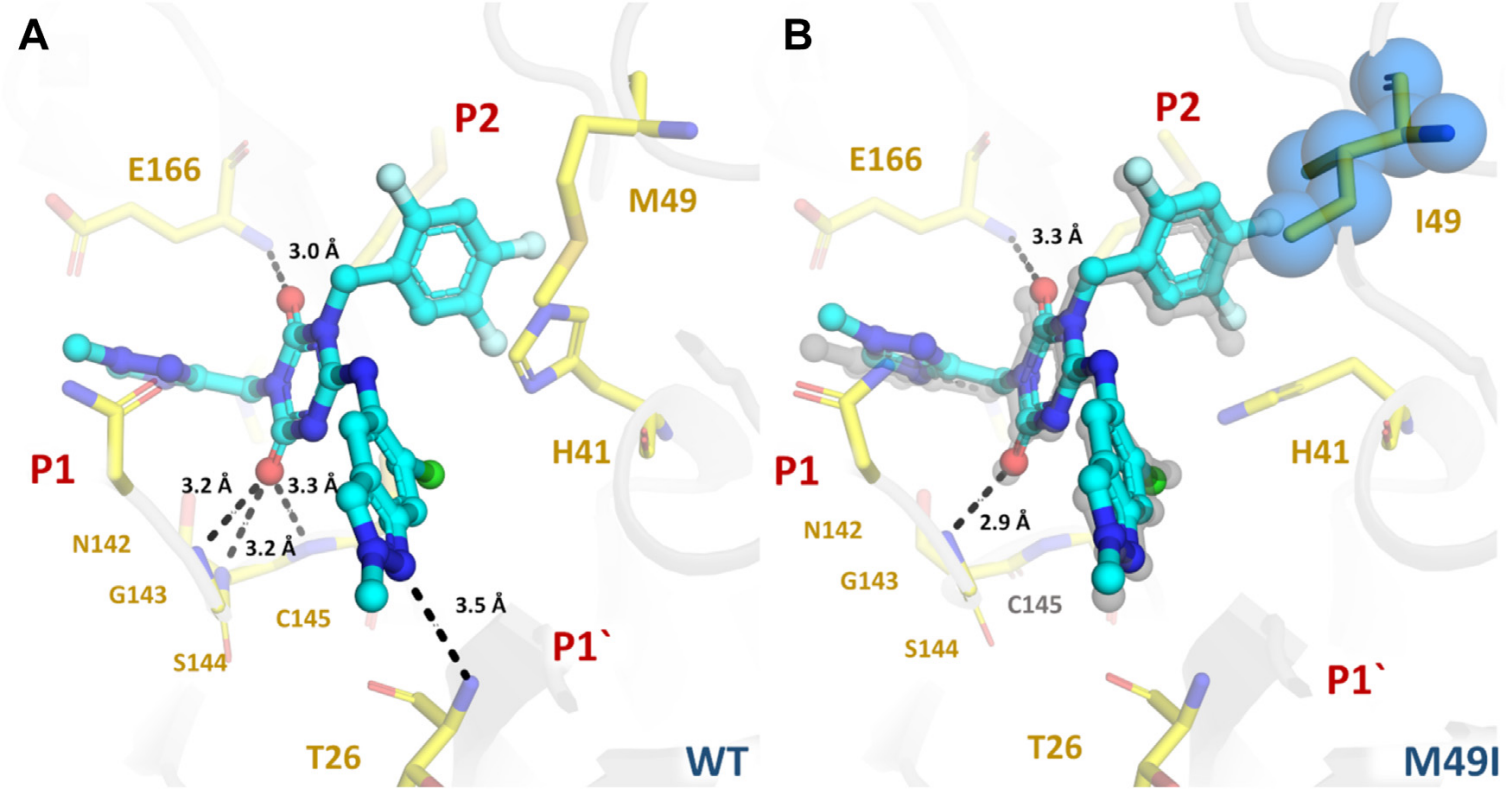
**Crystal structures of M**^**pro**^**in complex with ensitrelvir.** M^pro^ is displayed as *cartoon* and colored in *gray*; mutant residue is shown as *spheres* and colored in *blue*. Selected residues are displayed as *yellow sticks*. Ensitrelvir is shown as *ball* and *stick* and colored in *cyan*. Substrate-binding subsites are labeled in *red*. *A*, M^pro^ WT (PDBid 8DZ0). *B*, mutant M49I (PDBid 8DZ1) aligned with M^pro^ WT, displayed as *gray transparent sticks*. Polar contacts are showed as *black dashes*.

For ensitrelvir complexes, both WT and M49I structures exhibited a similar noncovalent binding mode maintaining the key interactions with residues T26 (3.5 Å), G143 (3.2 Å), S144 (3.2 Å), C145S (3.3 Å), H163 (3.1 Å), and E166 (3.0 Å) through productive hydrogen bonds (Fig. 6). A characteristic of ensitrelvir in complex with M^pro^ is the rotation of H41 relative to the apo/nirmatrelvir structure, forming a face-to-face π stack with the 3,4,5-trifluorobenzene moiety of ensitrelvir (Fig. 6). However, the crystal structure of the M49I mutant revealed that the substitution dislocated the ligand orientation within the binding site toward the P2 position. The binding mode analysis suggested that this displacement is related to the greater hydrophobicity and steric volume of the isoleucine residue in comparison with the methionine residue. In addition, for the WT structure, the side chain of the catalytic H41 residue is flipped undergoing a π-interaction with the trifluoro-phenyl substituent, whereas for the M49I mutant, the H41 rotamer is positioned similarly to M^pro^ apo structures, which causes the loss of hydrogen bonds between ensitrelvir and residues T26, S144, and C145 (Fig. 6).

## Discussion

We identified 15 active site polymorphisms within our searching criteria for nirmatrelvir and 13 for ensitrelvir (Fig. 1). None of these mutations are currently present in strains considered of interest by the World Health Organization due their low occurrence, but the broad use of nirmatrelvir/ensitrelvir could induce the selection of these variants. Here, we performed the kinetic characterization and evaluated the effects of these mutations in the inhibitory constants of nirmatrelvir and ensitrelvir. Our data show that most of the selected polymorphisms resulted in enzymatically efficient forms of M^pro^, indicating that these mutations would likely be able to generate versions of M^pro^ with high fitness for selection (Fig. 3). Only G143S, Q189K, and A193V mutants exhibited significantly decreased enzymatic efficiency against the fluorogenic substrate, which might hamper these polymorphisms’ capacity of being selected without any compensatory mutation.

Nirmatrelvir is a covalent peptidomimetic inhibitor of SARS-CoV-2 M^pro^ developed by Pfizer, designed to compete with the substrate at the P1 and P2 subsites (14). Nirmatrelvir key interactions with M^pro^ include hydrogen bounds with H163, E166, and Q189. A recent study used deep mutational scanning to map the mutational landscape of M^pro^ and found that M49, N142, E166, P168, Q189, and A191 residues have high flexibility, overlapping with most of our chosen residues based on real-world data, and found mutant E166V to be highly resistant to nirmatrelvir (23). In another recent prepublication, the authors used a similar approach to ours to identify 11 mutants resistant to nirmatrelvir, including S144M/F/A/G/Y, M165T, E166Q, H172Q/F, and Q192T/S/V (24). Also in a recent publication, authors followed resistance generation on more than 50 viral linages in presence of nirmatrelvir and identified 23 hotspots for mutation on M^pro^, including E166, R188, A191, and A193, with the highest resistance associated with mutant E166V (25). Another recent publication used a combination of *in silico* mutational scanning combined with *in vitro* testing to identify mutants N142L, E166M, Q189E, Q189I, and Q192T where nirmatrelvir showed reduced potency (26). Similar residues were also identified by *in silico* molecular dynamics and high-throughput protein design approaches (27, 28).

The mutant panel tested herein explored the capacity of nirmatrelvir to retain *in vitro* activity against circulating active polymorphisms of M^pro^. Our data indicated that nirmatrelvir showed nanomolar inhibitory activity against most of the tested mutants, including the most common mutation found, A191V, which showed a 2.6-fold increase in the *K*_i_ value for nirmatrelvir. The mutations that affect P2 subsite residues M49 and M165 had a minor effect on nirmatrelvir activity (Fig. 3*B*). The structure of M49I in complex with nirmatrelvir showed that the mutation has also a minor effect on ligand orientation relative to the WT, where all key molecular contacts were maintained (Fig. 4*D*). More distant mutations affecting subsites P3-P4 small residues, such as A191T, A191V, A193T, and A193S (but not A193V) have shown significantly increased *K*_i_ values for nirmatrelvir when compared with P1 and P2 mutations. The structure of A193T and A193S mutants showed that the increased steric volume and polar features of the side chains caused a displacement of the inhibitor related to the WT (Fig. 4, *E* and *F*). As expected, we also notice that less conserved mutations in a specific amino acid seemed to have a greater effect on inhibitory constants of compounds, as exemplified by R188K *versus* R188S (1.4- *versus* 2.9-fold increase in *K*_i_). Another potentially impactful resistance mechanism depicted here was the one caused by the Q189K mutant, in which the crystal structure revealed that the absence of a productive bond between the oxygen atom of the amide group of nirmatrelvir and the side chain of Q189 caused a rearrangement of the inhibitor’s binding mode (Fig. 4*B*), leading to an increase of 16-fold in the *K*_i_ value (Fig. 3*B*).

The mutation of G143 to serine shifted the charge distribution and/or stereochemistry on S1, preventing the stabilization of the pyrrolidinone group into a single conformation (Fig. 5). A similar effect might explain why this mutation also depleted the enzyme efficacy *versus* a substrate containing Gln side chain at P1. Despite the high decrease in inhibitory constants generated by this mutation, the low catalytic efficiency exhibited by this polymorphism might limit its ability to be selected over other strains, unless it is associated with secondary mutations that compensate the deleterious effect.

Ensitrelvir is a nonpeptidic noncovalent inhibitor of SARS-CoV-2 M^pro^, developed by Shionogi using an intense structure-based drug design program (12). Their clinical candidate uses a methyl-triazole substituent (P1) linked to a trifluorophenyl group (P2) by a triazinane-2,4-dione, which also connects a third substituent containing a 6-chloro-2-methylindazol-5-amine that competes with the P1′ subsite (12). This scaffold allowed ensitrelvir to achieve high enzymatic/antiviral activity and great metabolic stability, with key hydrogen bounds formed with T26, G143, H163, and E166 (12). In contrast with nirmatrelvir, ensitrelvir seems to be more susceptible to mutations affecting the P2 subsite, such as M49I and M49T (Table 1). The structure of M49I in complex with ensitrelvir revealed that the higher hydrophobicity of the isoleucine side chain caused a displacement of the inhibitor toward the P2 cavity, likely affecting its inhibitory activity (Fig. 6*B*). Another key difference between ensitrelvir and nirmatrelvir resistance profiles is that the former seems to retain near full activity against the Q189K mutant.

The combined results suggest that these two distinct inhibitors have a different resistance profile against a panel of mutants, which can be explained by the distinct binding modes to M^pro^. These results are important not only in the monitoring of emergence of resistant strains of SARS-CoV-2 but also for planning a more suitable treatment in the event of one of these polymorphisms becoming a strain of concern. Moreover, the depicted complexes between inhibitors and mutants help us to understand the structural features involved in resistance, which should assist the development of the next generation of M^pro^ inhibitors.

## Experimental procedures

### Identification of M^pro^ polymorphisms

We selected polymorphic versions of M^pro^ that have been already identified in circulation. To do this, we sorted sequencing data available from the GISAID hCoV-19/SARS-CoV-2 sequences database (22) in CoV-GLUE (http://cov-glue.cvr.gla.ac.uk) relative to M^pro^. Then, we selected all mutants that were within an 8.0-Å radius of the drugs and with n ≥ 10 individuals.

### Site-directed mutagenesis, protein expression and purification

Cloning and expression were performed as described in Noske *et al.* (7), 2021. The viral cDNA template (GenBank MT126808.1), kindly provided by Dr Edison Durigon (University of São Paulo), was synthesized using the SCRIPT One-Step RT-PCR kit (Cellco Biotec) and random hexamer primers. The M^pro^ coding region (residues 3264–3569) was inserted into the pET_M11/LIC vector using the ligation-independent cloning method (29). The final construct contains an N-terminal 6xHis-tag followed by a Tobacco Etch Virus site and the native N-terminal M^pro^ residues (YFQGAMSAVLQ↓SGFRK). For site-directed mutagenesis, the pET_M11/LIC-Mpro vector was used as template for inverse PCR (7). All the PCRs were performed using FastPol polymerase (Cellco Biotec) and primers from Table S1. The PCR product was digested with DPNI (NEB), followed by treatment with T4 Polynucleotide kinase (Thermo Fisher Scientific) and T4 DNA ligase (Cellco Biotec). Mutations were confirmed by sequencing.

We used a self-cleavable construct of SARS-CoV-2 M^pro^ that was successfully expressed and purified using ammonium sulfate precipitation, followed by anion-exchange chromatography, with a final yield of 2.5 mg l^−1^ of culture (7). The same expression and purification protocols were used to obtain M^pro^ mutants. All mutants were obtained with a similar elution profile and final yield, except for N142L, which did not have an expression level suitable for purification. For protein expression, *E. coli* BL21 cells containing the recombinant plasmids were grown in ZYM 5052 medium (30) until *A*_600_ reached 0.6. Protein expression was induced by reducing the temperature to 18 °C, and cells were grown for 16 h. Cells were harvested by centrifugation at 5000*g*, at 4 °C for 40 min and resuspended in lysis buffer (20 mM Tris pH 7.8, 150 mM NaCl, 1 mM DTT). Cells were disrupted by sonication. The lysate was clarified by centrifugation at 15,000*g*, at 4 °C for 30 min. After expression, M^pro^ was obtained in the native form after autocleavage. The protein was obtained by precipitation with addition of 1.5 M ammonium sulfate followed by incubation on ice for 10 min. The precipitated protein was isolated by centrifugation at 15,000*g* at 4 °C for 15 min. The protein was resuspended in lysis buffer and injected into a Superdex 200 26/100 column (GE Healthcare) pre-equilibrated with gel filtration buffer (20 mM Tris pH 7.8, 50 mM NaCl, 1 mM DTT). After size exclusion chromatography, the protein was buffer exchanged to 20 mM Tris pH 8.0, 1 mM DTT and further purified by ionic exchange chromatography using a Mono-Q column (GE Healthcare). The protein was eluted with a linear gradient of a buffer containing 20 mM Tris pH 8.0, 1 M NaCl, and 1 mM DTT. Fractions containing the purified protein were collected and quantified using the measured absorbances at 280 nm and the theorical extinction coefficient of 32,890 M^−1^ cm^−1^. Protein purity was analyzed by SDS-PAGE. For enzymatic assays, protein was aliquoted at 0.5 mg/ml and flash-frozen using liquid nitrogen. Samples were stored at −80 °C until use.

### Protein crystallization and soaking

SARS-CoV-2 M^pro^ mutants were crystallized using the sitting-drop vapor diffusion method. A volume of 1 μl of protein at 5 to 14 mg/ml was mixed with 1 μl of precipitating solution containing 0.1 M Mes pH 6.7, 8% PEG 4K, and 5% dimethyl sulfoxide (DMSO) (31) and 0.2 μl of seed stock. The seed stock was obtained from orthorhombic crystal system M^pro^ crystals (7). Crystals were observed after 1 to 2 days at 16 °C.

Crystals of SARS-CoV-2 M^pro^ mutants in complex with nirmatrelvir and ensitrelvir were obtained by soaking the compound into M^pro^ mutants apo crystals grown as described previously. A volume of 2 μl of a solution containing 80% PEG 400, 20% DMSO, and 10 mM of compound was added directly into the 2 μl crystallization drops. Crystallization plates were incubated at 16 °C for 24 h. Crystals were manually harvested and flash-cooled in liquid nitrogen for data collection.

### Data collection, processing, structure solving and refinement

Diffraction data collection was performed at MANACA beamline at the Brazilian Synchrotron SIRIUS using a Pilatus 2M detector (Dectris). Data were processed and scaled using autoPROC/STARANISO from Global Phasing (32, 33). Resolution cutoff was determined by CC_1/2_ (34, 35). For structure determination DIMPLE (36) was used for automated molecular replacement and initial refinement, using as initial template and search model the orthorhombic crystal structure of WT M^pro^ (PDBid 7MBG). Ligand and covalent link restraints were generated using AceDRG through the CCP4i2 program suite (37, 38). Refinement was conducted using REFMAC5 (39) or Phenix.refine, and manual rebuilding was performed using Coot (40). Structure validation was conducted using MolProbity (41). Figures were generated using Pymol (Schrödinger, LLC).

### Activity and inhibition assays

Ensitrelvir was purchased from TCG Lifesciences, whereas nirmatrelvir was kindly donated by Prof Carlos A. Montanari. All enzymatic assays were performed using fluorescence resonance energy transfer based substrate DABCYL-KTSAVLQ↓SGFRKM-E(EDANS)-NH2 in assay buffer (20 mM Tris pH 7.3, 1 mM EDTA, 1 mM DTT) in Corning 384-well white microplates. M^pro^ mutants were diluted to a final concentration of 40 nM. To determine the kinetics parameters (*K*_*m*_, V_max_, and k_cat_), the substrate was diluted to a range of concentrations from 200 μM to 0.1 μM. Reactions were previously incubated at 37 °C for 10 min and started by addition of substrate in the respective concentrations. Fluorescence measures were monitored in SpectraMax Gemini EM Microplate Reader with λ_exc_/λ_emi_ of 360/460 nm, every 60 s over 60 min at 37 ° C. The initial velocity was derived from the slope of linear phase of each time-course reaction, and Michaelis–Menten fitting was obtained using Origin Pro 9.5.1 Software (OriginLab). Relative efficiency of M^pro^ mutants was calculated by comparing the k_cat_^/^*K*_*m*_ relative to WT M^pro^. For IC_50_ determination, reactions containing nirmatrelvir or ensitrelvir from 10 μM to 0.0006 nM were previously incubated at 37 °C for 10 min and started by addition of 10 μM of substrate. Inhibition percentages were determined by comparison with the DMSO control. All assays were performed in independent triplicates, and presented values were determined from average values. For determining the *K*_i_ values, nirmatrelvir was considered an uncompetitive inhibitor, whereas ensitrelvir was considered a competitive inhibitor, and values were calculated using IC_50_-to-*K*_i_ (42).

## Data availability

Structure factors and atomic coordinates have been deposited with the Protein Data Bank with accession codes PDB IDs 8DZ2, 8E25, 8E26, 8DZ9, 8DZ6, 8E1Y, 8DZA, 8DZ0, and 8DZ1. Other data are available from the corresponding author upon reasonable request.

## Supporting information

This article contains supporting information.

## Conflict of interest

The authors declare that they have no conflicts of interest with the contents of this article.
